# Garlic in Tuberculosis

**Published:** 1915-02

**Authors:** 


**Garlic in Tuberculosis.** The "New Orleans M. & S. Jour.," states that a Dublin physician has used it with success and that it is being tried at the Metropolitan Hospital of N. Y. The active principle, Allyl sulphid forms about 1% of the juice. (Note: More than 25 years ago, Dr. Charles Cary called attention to the relative immunity of the garlic eating peoples.)

